# Comparison of residential and mobility-integrated air pollution exposures from tracking campaigns and agent-based modelling in Switzerland and the Netherlands

**DOI:** 10.1038/s41370-025-00836-5

**Published:** 2025-12-26

**Authors:** Kees de Hoogh, Benjamin Flückiger, Nicole Probst-Hensch, Ayoung Jeong, Medea Imboden, Aletta Karsies, Oliver Schmitz, Roel Vermeulen, Kalliopi Kyriakou, Aisha Ndiaye, Youchen Shen, Derek Karssenberg, Danielle Vienneau, Gerard Hoek

**Affiliations:** 1 Swiss Tropical and Public Health Institute, Allschwil, Switzerland; 2 University of Basel, Basel, Switzerland; 3 Department of Physical Geography (Geo), Utrecht University, Utrecht, the Netherlands; 4 Institute for Risk Assessment Sciences (IRAS), Utrecht University, Utrecht, the Netherlands

**Keywords:** GPS tracking, Air Pollution, Agent based modeling, Mobility, Residential

## Abstract

**Background:**

Studies investigating the health effects of long-term exposure to air pollution generally rely on the outdoor air pollution exposure assigned at the residential address. By ignoring time activity, population exposure misclassification could potentially lead to loss of precision or bias in epidemiological studies.

**Objective:**

We aimed to assess how residential-based air pollution exposures compared with “real” tracking-based exposures.

**Methods:**

We conducted two tracking campaigns in Switzerland (CH) and the Netherlands (NL) with 686 participants followed for 2 weeks with GPS trackers whilst keeping time activity diaries. In addition, we simulated mobility and commuting tracks for the same subjects using agent-based modeling (ABM) with information from census and travel survey data to estimate mobility-integrated air pollution exposures. Exposures were calculated by overlaying residential address, measured (GPS) and modeled (ABM) tracks with annual average hourly NO_2_ and PM_2.5_ concentration surfaces.

**Results:**

We found strong agreements between residential and tracking-based exposures in CH for both pollutants (*R*^2^ > 0.76) and NL for NO_2_ (*R*^2^ = 0.79), and weaker agreement in NL for PM_2.5_ (*R*^2^ = 0.56). Similarly, the agreement between ABM and tracking-based exposures was strong for NO_2_ (*R*^2^ > 0.77 in CH and NL), while for PM_2.5_ it was stronger in CH (*R*^2^ = 0.80) than in NL (*R*^2^ = 0.54). The highest correlations were between residential and ABM exposures (*R*^2^ > 0.96 for both pollutants). Using information commonly available even in large administrative cohorts, we found that exposures derived from the tracking campaigns agreed well with ABM in our two study areas.

**Significance:**

Our study supports the use of residential exposures in epidemiological studies on long-term health effects of air pollution, whilst acknowledging that ABM, especially if the work location is known, can be a useful tool to estimate mobility-integrated exposures.

**Impact statement:**

- Our research supports the use of residential exposures in studies investigating the long-term health effects of air pollution, whilst acknowledging that agent-based modeling, especially if the work location is known, is valuable for estimating mobility-integrated exposures. Our findings are broadly applicable to air pollution epidemiology, in particular, studies of large populations that rely on exposure modeling.

## Introduction

Air pollution has been associated with various adverse health effects, including mortality.[1] A recent WHO report reported an estimated 4 million deaths annually due to long-term ambient air pollution globally [2–4]. In the Global Burden of Disease assessments, air pollution ranks as the most influential environmental exposure [5]. The evidence for these burden of disease assessments was primarily derived from epidemiological studies, especially on long-term air pollution exposure. A Panel appointed by HEI recently reviewed epidemiological studies of traffic-related air pollution (TRAP), addressing exposure assessment issues in detail [6–8]. The Panel primarily assessed how well specific methods assessed the outdoor concentration of TRAP, considering the spatial alignment of outdoor exposure as assigned to the residential address, for example, by assessing the spatial resolution of the address and exposure surfaces. Virtually all studies assigned outdoor concentrations to the residential address only [6, 7].

Studies investigating the associations between air pollution and health have shown that real personal exposure depends on the time spent in specific micro-environments [9, 10]. These real personal exposures to air pollution can be assessed directly, for example, by personal monitoring, or indirectly by combining time activity data with concentrations measured or estimated in important micro-environments[9]. Direct or indirect assessment of personal exposures is typically not used in large epidemiological studies. Direct assessment is too costly and logistically challenging due to the large numbers necessary to detect the effect of long-term air pollution on health. It is also not feasible to carry out indirect assessment because large studies, including studies using administrative databases [11, 12], only have information on the people’s home address and typically do not collect time activity data. There are some studies, including the Dutch PIAMA birth cohort and the Swiss SAPALDIA studies, set up specifically to investigate the health effects of air pollution, where information on the work and school addresses had been collected [13, 14].

Epidemiological studies on air pollution and other environmental exposures have been criticized for not taking into account time-activity patterns [15, 16]. Whilst it is understandable that epidemiological studies have relied on the residential address, the question remains as to how well exposure is assessed by focusing on the residential address only.

A comprehensive review of studies comparing residential-based air pollution exposures with time-activity integrated air pollution exposures showed that residential address-based air pollution exposure and mobility-integrated exposure were mostly highly correlated, but the number of studies is still limited [17]. Furthermore, studies used different methods to assess mobility-integrated exposure, and agreement differed somewhat, related to commuting patterns in the population. The few studies that have assessed time activity patterns and related mobility-integrated air pollution exposures are typically either empirical tracking studies in selected and often small study populations [18–20] or Agent-Based Modeling (ABM) studies that can be applied to larger populations based on time survey data [21, 22].

In this paper, we apply both approaches (GPS tracking and ABM) to investigate whether more sophisticated estimates of individual air pollution exposure, considering population mobility, are different from estimates based on the residential address location only. To achieve this, we use data collected in two purpose-designed tracking campaigns conducted in Switzerland (CH) and the Netherlands (NL), as part of the “Accounting for MOBility in AIR pollution exposure estimates in studies on long-term health effects” (MOBI-AIR) study. To assess the robustness of the findings, we performed the study in two countries with different commuting patterns. We captured mobility data of almost 700 participants using GPS trackers and time-activity diaries. In addition, we simulated mobility and commuting tracks for the same sample in both countries using ABM. The measured and modeled time-activity data were combined with detailed spatial-temporal air pollution data to enable calculation of residential, mobility integrated (using ABM) and tracking-based NO_2_ and PM_2.5_ exposure estimates for the individuals. These resulting exposures were then compared.

## Materials and methods

### Tracking campaign

We conducted tracking campaigns during 2022/23 in the region of Basel (cantons of Basel-Stadt and Basel-Landschaft), Switzerland and across the Netherlands (mainly in the province of Utrecht), including 489 Swiss and 189 Dutch participants, collecting detailed information on their mobility patterns over a 2-week period using a purposely designed mobile phone study app and a GPS tracker [23]. The tracking campaigns included a baseline questionnaire and a time activity diary (TAD). The existing population-based COVCO-Basel cohort [24] was used for embedding this study and for recruitment in the Basel region (Switzerland), achieving a total of 489 participants (recruitment rate of 33% from the total of 1475 people invited by email). In the Netherlands, we used a combination of random sampling from the whole Dutch population and a more targeted approach, distributing leaflets around Utrecht, adding a 25 Euro voucher as an incentive, reaching a total of 189 participants.

Participants were asked to download the study app, in which they filled in a daily TAD during the 2 weeks of the tracking campaign. The study app also collected their location information every 3–4 min via the mobile phone integrated GPS. In addition, we asked the participants to carry a high-precision GPS tracker (location time stamp every 20 s) specifically developed for the study. Informed consent was obtained from all subjects.

Location points from the study app and the GPS tracker were combined and linked to both the corresponding TAD information and air pollution concentrations extracted from long-term hourly weekdays and weekends air pollution raster files (see Section “Exposure estimates”).

In the baseline questionnaire, participants were asked for details on the environment around their home, neighborhood, commuting behaviors, general health status, and sociodemographic aspects. Employed participants were asked to provide their work address information to assess the accuracy of the ABM analysis.

### ABM

The ABM applied in this study has been described in detail elsewhere [25, 26] following the approach of Lu and Schmitz [21]. Depending on the profile of an individual (i.e., agent), an activity diary was created, which, starting from the home location, simulated activities such as commuting, work, shopping, and recreation, including the time spent at these locations and activities. Based on the Euclidean distance between the home and simulated work locations, the commuting mode (i.e., walk, bike, car, and public transport) was randomly determined, and the shortest route was subsequently calculated across the transport network. For each agent, a Monte Carlo simulation was applied to calculate 50 realizations, meaning that 50 random work locations were identified per agent, leading to 50 corresponding routes. The locations and routes were overlaid with hourly air pollution data, with different diurnal patterns for weekdays and weekends reflecting traffic flows, to calculate aggregated air pollution exposures. For each agent, the mean and standard deviation of air pollution concentrations of the 50 realizations were calculated. A simulation was performed comparing the mean of the distribution of the 50 ABM realizations vs. an assignment of a single random realization. Finally, for the subgroup of participants with known work address information provided in the baseline questionnaire in Switzerland and the Netherlands, we also performed ABM modeling, taking the work location into account.

Three main profiles were created for the agents: a residential, a homemaker and a commuter profile. The residential profile assumed the same activity during weekdays and weekend days for 24 h at the residential address location. The homemaker represented the non-working population with three sub-profiles simulating activities in an increasing activity space (1, 5 and 10 km buffer around residential address), with the same activity pattern during weekdays and weekend days. Lastly, the commuter profile consisted of 9 sub-categories (all, male, female, male (low, middle, high SES) and female (low, middle, high SES)). Commuting patterns for the different commuter profiles were based on the country-specific origin-destination matrices. In the Netherlands, we obtained data from the National Travel Surveys from Statistics Netherlands [27] covering the years 2011–2019. These annual surveys provide information on the daily mobility behavior of a sample of 40,000 randomly selected Dutch households. For each household, the survey asked details for one specific day, about the number of trips, geographical location and times of start and end for each trip and mode of transport. In Switzerland, we used origin-destination matrix data from the Structural Survey, a component of the census, which is based on interviews of 200,000 people each year. The Survey collected individual-level data covering similar details as in the Dutch survey, also for the period 2010–2019 [28]. The work location was randomly drawn in a destination municipality. Each participant in the campaign was designated the profile that best fit their demographic data collected in the baseline questionnaire.

### Exposure estimates

Two different approaches were used for the exposure modeling. In Switzerland, we rescaled annual average PM_2.5_ and NO_2_ surfaces (100 × 100m) for the year 2016, from previously published spatiotemporal geostatistical models [29, 30] to long-term hourly weekday and weekend day surfaces following a method previously applied by de Nazelle et al. [31]. Measured daily PM_2.5_ and NO_2_ data respectively from 9 and 63 background monitoring stations were used to calculate ratios that were then applied to the long-term concentration surfaces using the formula:

1 $${{Ratio}}_{{annual}-{hr}}={C}_{{BS}-{hr}}/{C}_{{BS}-{annual}}$$

Where: *Ratio*_*Annual-hr*_ is the ratio of the air pollutant concentration measured at background monitoring station (BS) at time hr (hour 1 to 24 h averaged over a year, hour 1 to 24 h averaged for weekday and weekend); *C*_*BS-hr*_ is the measured air pollution concentration averaged for each given hour per year; and *C*_*BS-annual*_ is the annual average air pollution concentration at all background stations in Switzerland.

These diurnal ratios were then applied to the annual average air pollution surfaces using the following formula:

2 $${C}_{P-t}={C}_{P-{annual}}* {{Ratio}}_{{annual\_t}}$$

Where: *C*_*P-t*_ is the time-adjusted prediction at point *P* and time *t*; and *C*_*P-annual*_ is the annual average pollutant concentration predicted by the LUR model at point *P*.

For the Netherlands, Ndiaye et al. [32] developed maps of annual average hourly concentrations of PM_2.5_ and NO_2_ using land use regression models (supervised linear regression (SLR) and random forest (RF)) based upon air quality monitoring data (*n* = 544 for NO_2_; *n* = 227 for PM_2.5_) in the Netherlands and the neighboring countries Belgium and Germany. Spatial variations of the average hourly concentrations were modeled for the years 2016–2019 combined, for two seasons (cold and warm), and for two weekday types (weekend and weekdays). Predictor variables included information about land use, roads, meteorology, population and satellite retrievals and chemical transport model estimates. SLR and RF models performed similarly, with 5-fold cross-validation *R*^2^ for the hourly models between 0.50–0.78 and 0.24–0.62 for NO_2_ and PM_2.5_, respectively. For the purpose of this study, we used the hourly surfaces derived from the RF models.

**Fig. 1:**
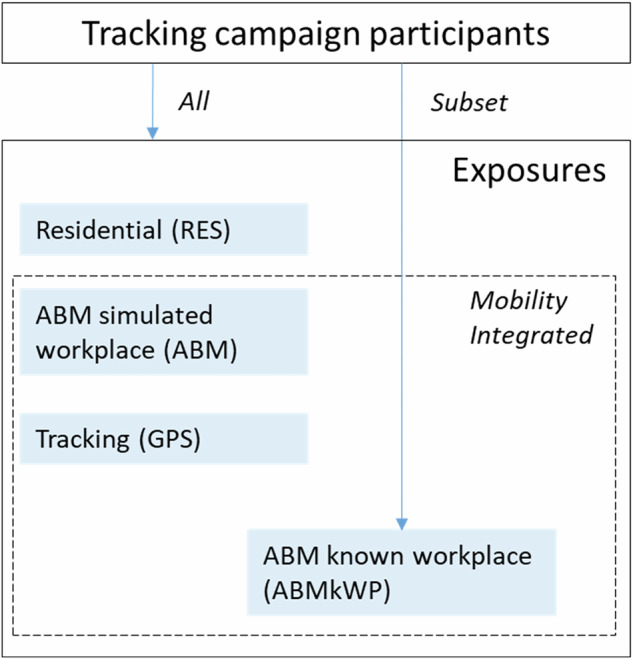
Overview of different air pollution exposures calculated for tracking campaign participants. Schematic representation of the different air pollution exposures calculated for the tracking campaign participants in Switzerland and the Netherlands; for all participants, Residential (RES), mobility-integrated ABM (with simulated workplace) and GPS exposures; for a subset, also the mobility-integrated ABMkWP (with known workplace).

Figure 1 shows a diagram with the different exposures calculated for the tracking campaign participants in both countries.

### Statistical analysis

Residential (RES), mobility-integrated ABM simulated workplace (ABM) and tracking (GPS) exposures were evaluated on their agreement through Bland-Altman plots, and further compared using the coefficient of determination (*R*^2^) and visualized in scattergrams. For the purpose of this assessment, we defined correlation coefficients (*R*^2^) as weak (<0.16), moderate (>0.16–0.36), moderately strong (>0.36–0.64), strong (>0.64–0.81) and very strong (>0.81). All analyses were performed separately for the Basel and the Netherlands populations. Additionally, we compared ABM (using the mean of the 50 realizations) and ABM with a known workplace (ABMkWP). This analysis was performed on a subset of the participants from whom we received the work address from the baseline questionnaire (Fig. 1). Shortest routes were calculated from home to the known work locations, and exposures extracted. We also conducted a simulation comparing the mean of the 50 realizations with a single random exposure from the distribution of the 50 realizations.

## Results

### Tracking campaign populations

In both tracking campaigns, female participants were notably overrepresented (Table 1). The majority of participants were employed, while a smaller proportion consisted of retired individuals. Participants with higher levels of educational attainment and in elevated income brackets were also disproportionately represented. When compared to national demographic statistics, it is evident that the samples derived from the campaigns do not fully reflect the broader population structures of the respective countries. Participants of the tracking campaigns in both countries resided in more urbanized areas compared to the national population, with urbanicity scores [33] of 49.3 and 42.2 for the Swiss and Dutch campaigns, compared to 23.9 and 26.6 for the total Swiss and Dutch populations, respectively. Nonetheless, the campaigns captured a reasonable degree of variation, including individuals from younger and older age groups, participants with low to medium education levels and income, and those not engaged in formal employment. Additionally, we compared the residential-only air pollution exposures in the tracking campaigns with those in 2 cohorts used in our epidemiological pape [25]. For Switzerland, we used the Swiss National Cohort, which is a cohort based on the national census, from which we used those over 30 years for whom we could assign exposure (approximately *n* = 3.5 M). In the Netherlands, the EPIC-NL cohort was used (*n* = 33.475). In Switzerland, the tracking campaign has higher mean NO_2_ and PM_2.5_ exposures compared to those of the SNC (NO_2_, 22.25 vs 17.00 µg/m^3^; PM_2.5_, 15.05 vs 13.51 µg/m^3^), confirming the urban setting in the Swiss tracking campaign (Table 2). The comparison in the Netherlands shows almost identical residential-only exposure for the tracking campaign and the EPIC-NL cohort.

**Table 1:** Demographics of participants in the two tracking campaigns (percentages in each category calculated for the total population).

		Switzerland				The Netherlands			
		Track	%	National^1^	%	Track	%	National^2^	%
*N*		489		8,815,385		189		17,590,672	
Sex	Male	195	40	4,379,953	50	84	44	8,745,468	50
	Female	293	60	4,435,432	50	103	54	8,845,204	50
	Other	1	0			2	1		
Age	18–40	90	18	2,465,145	28	55	29	4,487,841	36
	40–60	268	55	2,513,375	29	71	38	3,415,571	27
	>60	131	27	2,253,637	26	63	33	4,683,950	37
Yearly	<36,000 CHF/<25,000€	22	4	1,615,440	18^3^	18	10	6,134,100	35
Gross	36–72,000/25–50,000€	86	18	1,681,480	19^3^	44	23	4,959,000	28
Income	>72,000 CHF/>50,000€	348	71	1,732,280	20^3^	100	53	2,273,300	13
	No answer	33	7			27	14		
Education	Pre-high school	125	26	706,950	15	18	10	1,959,000	20
	High school	34	7	2,167,980	46	14	7	3,720,000	38
	Higher Education	336	69	1,838,070	39	157	83	4,007,000	41
Employment	Fulltime	291	60	2,961,000	34	97	51	5,064,000	29
	Part-time/Irregular	116	24	1,752,000	20	37	20	4,673,000	27
	Homemaker/Not Working	27	6			16	8		
	Retired	44	9	2,115,692	24	36	19	3,578,513^4^	20
	Other/No Answer	11	2			4	2		
Type of area	Urbanicity^5^	49.29		23.86		42.19		26.57	

^1^www.bfs.admin.ch; data for 2022.

^2^ www.cbs.nl; data for 2022 (expect for income–data from 2020).

^3^https://de.statista.com/statistik/daten/studie/291841/umfrage/einkommensverteilung-in-der-schweiz/ Verteilung der Beschäftigten in der Schweiz nach Netto-Lohnhöhenklassen im Jahr 2020, Bundesamt für Statistik.

^4^www.svb.nl; data for 2022.

^5^de Hoogh et al. [33].

**Table 2:** Comparison of residential only NO_2_ and PM_2.5_ exposures (µg/m^3^) for the tracking campaign and cohort (SNC and EPIC-NL) populations showing mean and standard deviation (SD).

		Switzerland		The Netherlands	
		NO_2_	PM_2.5_	NO_2_	PM_2.5_
Tracking campaign	mean	22.25	15.05	25.47	12.93
	SD	9.20	2.72	4.70	0.48
Cohort		SNC		EPIC NL	
	mean	17.00	13.51	25.41	12.81
	SD	7.61	2.85	4.80	0.42

In the Dutch campaign, 22 of the initial 189 participants dropped out due to either problems with activating the GPS tracker or its being non-functional during the data collection period, leaving 167 participants with collected tracking data.

### Comparison of ABM simulated workplace and tracking campaign exposures

**Fig. 2:**
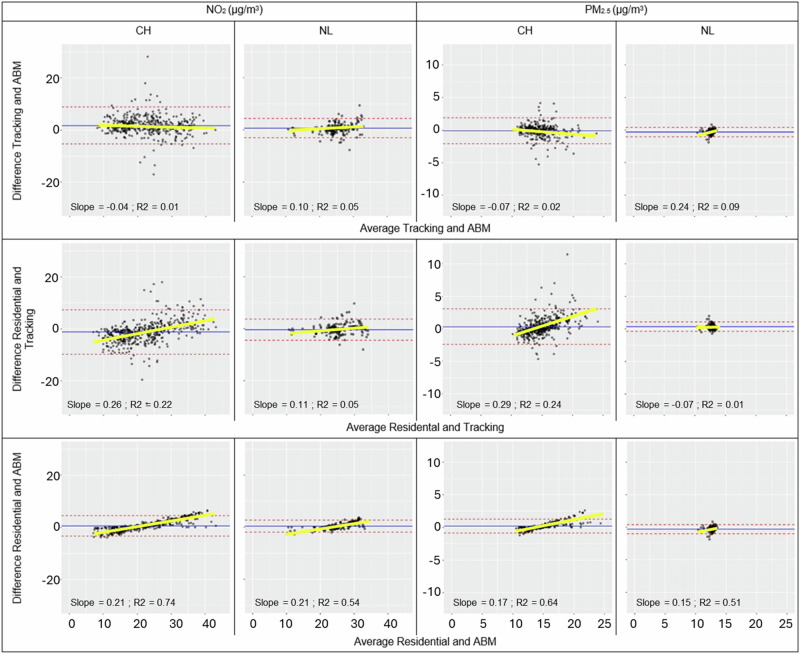
Agreement between the different air pollution exposure metrics using Bland Altman plots. Bland Altman plots showing comparisons between exposures for the Swiss and Dutch tracking campaign participants NO_2_ and PM_2.5_ (μg/m^3^) exposures based on the tracking data (GPS); ABM using simulated workplace (ABM) and the residential location only (RES). Blue line shows the mean difference, dotted red line 95% confidence limits for the difference, and yellow line the regression line, accompanied by slope and *R*^2^.

**Fig. 3:**
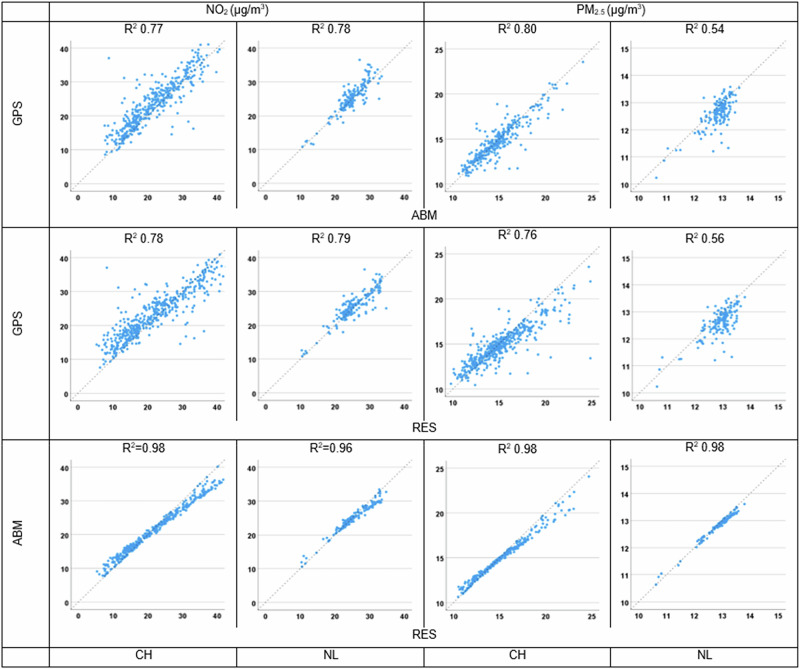
Relationship between air pollution exposures for Swiss and Dutch participants in the full dataset. Relationship (plus R^2^ s) between exposures for the Swiss and Dutch participants NO_2_ and PM_2.5_ exposures based on the tracking data (GPS), the ABM simulated workplace (ABM), and the residential location only (RES). x = y line is the dashed line.

We pairwise compared exposures derived from the tracking campaign (GPS), the ABMs simulated workplace (ABM) and known workplace (ABMkWP) and the exposures based on the residential location only (RES). Figure 2 shows the Bland Altman plots for the three NO_2_ and PM_2.5_ exposure comparisons (GPS vs. ABM; RES vs. GPS; RES vs. ABM), where each point represents a participant of the campaign. Figure 3 shows the corresponding scatter plots, including the correlation. In Table 3, exposure distributions of the different methods are shown for the two tracking campaign populations, including for a subset of participants for which the workplace location was known and where we could additionally compare exposures estimated by ABM with ABMkWP. For 86 participants in Switzerland, we were unable to assign a PM_2.5_ exposure as some routes and/or work locations from the 50 realizations calculated by ABM fell outside the Swiss PM_2.5_ modeling extent (i.e., Basel is a city lying on the border with France and Germany with frequent cross-border traffic).

**Table 3:** NO_2_ and PM_2.5_ exposure distributions (µg/m^3^) for the Dutch and Swiss tracking campaign for residential (RES), ABM simulated workplace (ABM) and tracking (GPS) exposures and additionally for a subset of participants with known workplace (ABMkWP).

		*n*	mean	SD	per25	median	per75	IQR
**The Netherlands**								
NO_2_	RES	167	25.47	4.70	23.01	24.86	28.94	5.93
	ABM	167	25.02	3.91	23.04	24.86	27.97	4.93
	GPS	167	25.57	4.43	23.40	25.37	28.31	4.91
	ABMkWP (subset)	89	26.18	3.39	23.79	26.36	28.77	4.99
PM_2.5_	RES	167	12.93	0.48	12.78	13.05	13.16	0.38
	ABM	167	12.86	0.43	12.76	12.97	13.07	0.32
	GPS	167	12.64	0.51	12.40	12.72	12.95	0.55
	ABMkWP (subset)	89	12.98	0.38	12.87	13.05	13.16	0.30
**Switzerland**								
NO_2_	RES	408	22.25	9.20	14.68	20.89	28.62	13.94
	ABM	408	21.65	7.46	15.93	20.76	26.96	11.03
	GPS	408	23.39	7.16	17.66	23.06	28.24	10.59
	ABMkWP (subset)	268	23.91	7.20	18.32	23.44	29.27	10.95
PM_2.5_	RES	322	15.05	2.72	13.07	14.68	16.54	3.47
	ABM	322	14.86	2.29	13.26	14.64	15.97	2.72
	GPS	322	14.74	2.13	13.24	14.49	15.88	2.64
	ABMkWP (subset)	201	15.13	2.06	13.73	14.85	16.40	2.68

*n* number of participants, *SD* standard deviation, *per25* 25th percentile, *per75* 75th percentile, *IQR* inter quartile range.

The Bland-Altman plots (Fig. 2) show that the mean differences between the three exposure metrics are small, and that for the majority of the points, the difference between exposure metrics is small. For some participants, fairly large differences are observed, especially when comparing tracking-based exposures with RES or ABM. The comparison of RES versus ABM for both NO_2_ and PM_2.5_ shows a systematic bias with *R*^2^’s of the regression lines between 0.51 and 0.74, and a positive slope (0.15–0.21) in both Switzerland and the Netherlands. At low concentrations, the ABM exposure was higher than the RES exposure and vice versa at high concentrations. This can be explained by the fact that participants who reside in a low-polluted area tend to have a higher overall exposure when mobility patterns are included (i.e., ABM), as the probability that they work in a higher-polluted area is increased. The opposite is observed for participants residing in a highly polluted area. The same pattern, although less strongly, can be seen in the comparison between RES and GPS, especially in Switzerland (*R*^2^ 0.22–0.24 and slope 0.26–0.29). The Bland-Altman plots for the comparison between GPS and ABM do not show a clear systematic pattern of differences versus the mean exposure, meaning that the estimated concentrations from the two approaches are similar.

Strong correlations were found between the GPS and ABM exposures for NO_2_ (CH: *R*^2^ = 0.77; NL: *R*^2^ = 0.78) and moderate strong for PM_2.5_ (CH: *R*^2^ = 0.80; NL: *R*^2^ = 0.54) (Fig. 3). The relatively lower correlation for PM_2.5_ found in the Netherlands is likely due to the small range of exposures (between 11 and 14 μg/m^3^) making it more difficult to estimate agreement. Similar correlations were found in the comparison between GPS and RES-based exposures (NO_2_: *R*^2^ = 0.78–0.79; PM_2.5_ *R*^2^ = 0.56–0.76). The comparison between ABM and RES yielded the highest correlations of >0.96 for both NO_2_ and PM_2.5_.

### Comparison with ABM known workplace

**Fig. 4:**
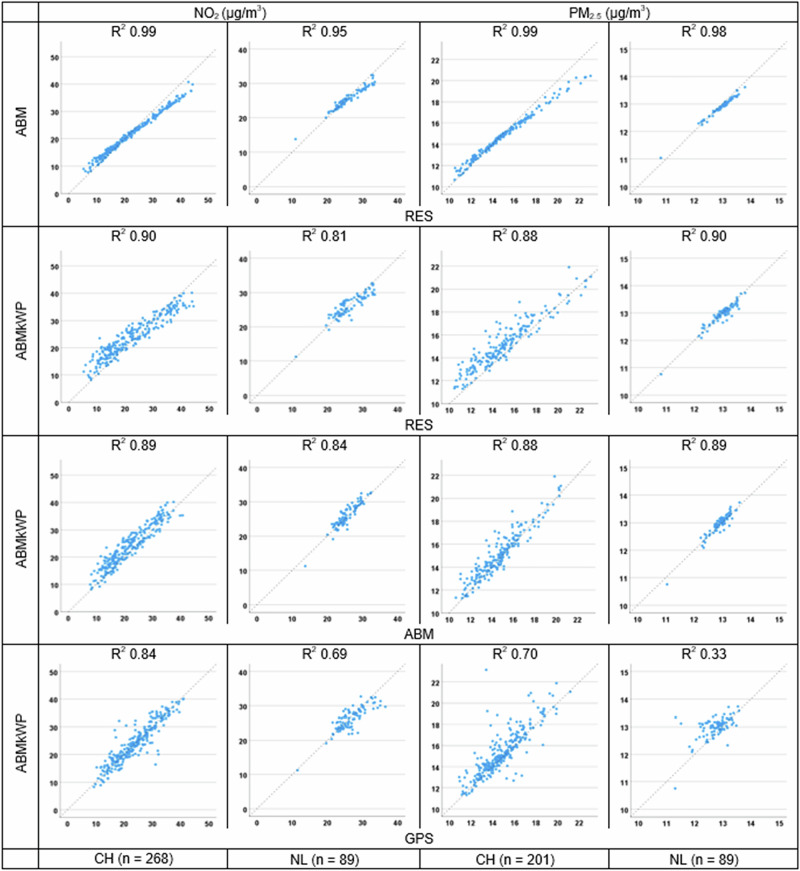
Relationship between air pollution exposures for Swiss and Dutch participants in the subset. Relationship (plus R^2^ s) between exposures for the Swiss and Dutch participants NO_2_ and PM_2.5_ exposures based on RES, ABM, ABMkWP and GPS NO_2_ and PM_2.5_ exposures in subsets of Swiss and Dutch tracking campaigns. *x* = *y* line is the dashed line.

For a subset of participants in both tracking campaigns (268 for NO_2_ and 201 for PM_2.5_ in CH, and 104 for both pollutants in NL), their work address information reported in the baseline questionnaire was taken into account in the exposure assessment. For both pollutants and both study areas, correlations between RES and both ABM exposures were strong to very strong (Fig. 4). The RES vs. ABMkWP correlations were slightly lower compared to those between RES and ABM exposures where the work location was probabilistically assigned (see Fig. 4). Correlations between the two mobility-integrated ABMs (ABM and ABMkWP) exposures were also very strong (*R*^2^ between 0.84 and 0.89), suggesting that ABM does a decent job in simulating the work address. Additionally, the very strong correlations between RES and ABMkWP (*R*^2^ between 0.81 and 0.90) give more weight to the robustness of the residential exposure. Lastly, we compared ABMkWP with GPS exposures. Similar to when using the full population (see Fig. 3), correlations were lower compared to the other three comparisons. The scatterplots illustrate higher variability in ABM modeled exposures with the known workplace compared to the simulated workplace, which includes averaging over 50 possible work addresses. The scatterplots of RES vs ABMkWP (Fig. 4) look more similar to the RES vs. GPS (Fig. 3) than the scatterplot of RES vs ABM. This is because both tracking and ABM know workplace use a specific track and work location for each individual, resulting in a similar “spread” in exposures between persons.

### Simulation random instead of the mean of 50 realizations

To further evaluate the effect of taking the mean of 50 realizations of ABM simulated workplace in our main analysis on the agreement between RES and ABM exposure, we performed a simulation with the assignment of a single realization from the distribution of the 50 ABM realizations (Fig. S1). For the comparison with residential exposure, correlation using the mean of 50 ABM (also shown in Fig. 3) had a higher correlation than when using the single draw for all pollutant/country combinations. For example, the correlations for NO_2_ were reduced from 0.99 to 0.93 and from 0.95 to 0.78, respectively, in Switzerland and the Netherlands. The points in the scatterplot show more variability around the 1:1 in the single draw comparison. The resulting scatterplots are more similar to the ABM known workplace comparisons, but now for the full tracking population.

## Discussion

We compared air pollution exposures based on the residential address with mobility-integrated air pollution exposures for almost 700 subjects in two tracking campaigns. For mobility-integrated exposures, we modeled exposures using ABM and assigned exposures as determined by the movements of individuals collected using the tracking data. In both tracking campaigns, conducted in two different study areas, very strong correlations were found between annual average residential exposure and mobility-integrated exposure for NO_2_ and PM_2.5_, both based upon observed tracking activity data and modeled ABM activity data. Furthermore, based on the same underlying air pollution data, the exposure levels—determined by the routes and time spent in specific locations—were very similar between both approaches. We did find a slightly smaller exposure contrast for ABM exposures (e.g., IQR = 4.93 µg/m^3^ for NO_2_ in NL; 11.03 in CH) compared to RES exposures (e.g., IQR = 5.93 µg/m^3^ for NO_2_ in NL; 13.94 in CH) (see Table 3). This can be explained by the phenomenon that subjects living in residential locations with high concentrations have a high probability of working in areas with lower concentrations and vice versa. Finally, we found that the residential exposure also correlated well with the mobility-integrated exposure based on actual time activity data in the tracking population.

### Exposures estimated from tracking and ABM with known and simulated workplace

The two approaches used in this study, ABM and tracking, differ in multiple aspects. ABM is a modeling approach that can be applied to large populations. As ABM is based on large-time activity surveys, it is more representative of the total population than tracking studies. ABM, however, makes assumptions about activity patterns, e.g., that activity can be represented by a simple standard pattern (for weekdays, for weekend days), neglecting short-time variations. It also assumes that within “profiles”, activities are comparable between individuals. Tracking relies on actual measurements and can therefore only be applied for short periods in smaller populations. When tracking is used for estimating long-term exposures, we must assume that the activity over the duration for which tracks are available is representative of the long-term activity of individuals. It is likely that actual tracks over a year would show substantially more variation than what is measured over a short time span, but the general pattern of activity (home, work, commute/on roads) is possibly quite well represented. In both approaches, sensitive populations like the ill and elderly are likely not well represented; however, as these people tend to stay more at home, we likely underestimate the correlation for these groups.

The high correlation between tracking and ABM-based exposures supports the use of ABM modeling in large populations. The Bland-Altman plots comparing these exposures were very stable for both pollutants in the two study areas, meaning that the tracking and ABM-based concentrations were similar. However, the Bland-Altman plots do show a bias in that participants of our tracking campaigns residing in areas with relatively low ambient air pollution may experience elevated mobility-integrated exposure due to an increased likelihood of commuting to or working in regions with higher pollution levels. Conversely, participants residing in highly polluted areas are less likely to travel to environments with significantly higher pollution concentrations, resulting in comparatively lower total exposure when mobility is accounted for. For our study areas, however, we can conclude that air pollution exposures derived from the tracking campaigns were successfully modeled with ABM, which used information commonly available in cohorts and larger administrative cohorts (e.g., age, sex, socio-economic status and employment status). The lack of a gold standard for evaluating long-term personal exposure to outdoor-generated pollution complicates the assessment of how well the three exposure measures reflect true personal exposure.

ABM with simulated work location exposures, by design, also has more uncertainty compared to ABM with known work location. In addition, differences with residential exposures are smaller for ABM with a simulated work address. In general, ABM with a known work location is preferable and collecting work location data in cohort studies is advantageous. The high correlation between the two ABM approaches and the good correlation with tracking-based exposures, however, suggests that ABM with simulated work addresses provides a reasonable approach to estimate exposure beyond residential address in studies that do not have known work addresses.

### Comparison with previous studies

Our observations are in line with several previous studies comparing residential and mobility-integrated exposures [13, 14, 34–36]. In all these studies, agent-based modeling was conducted using time surveys. Setton and co-workers documented that the agreement between residential and mobility-integrated exposure diminished with increasing time at work and with increasing distance between home and work locations [36]. A recent study using tracking data also reported high correlations for noise and PM_2.5_ [20]. Other studies have performed comparisons without ABM, thus based solely on exposure determined at the residential address location (home) vs. exposures including work address (work) and/or during commute. A study in Basel, Switzerland, compared exposure at the residential address with exposure whilst commuting and at the work/school address [14], indicating that, while there is room for improvement, it is reasonable to use exposure characterized at the residential address. A study in Montreal, Canada, showed that almost 90% of individuals had a lower 24-h daily average NO_2_ estimated at home compared to a mobility-integrated NO_2_ exposure [37]. Researchers in the Netherlands followed 269 adults with a GPS-enabled App for 7 days and compared the residential exposure only with a mobility-integrated PM_2.5_ exposure, concluding that the residential exposure was a good proxy for overall exposure to outdoor air pollution [20]. A study in Shenzhen, China, used cell phone data from more than 300,000 individuals to assess the impact of mobility on air pollution exposure. They concluded that while mobility impacted exposure on the individual level, it did not significantly impact exposures at the population level, in particular for larger studies [38]. A study in the UK compared population-weighted NO_2_, PM_2.5_ and O_3_ exposures at the residential address only with a combined residential and work exposure. They used a chemistry transport model to estimate rush-hour specific long-term averages and found only a small increase in population-weighted NO_2_ and PM_2.5_ exposures when including the work location (2 and 0.3%, respectively) [39].

Few studies have applied ABM to assess mobility-integrated air pollution exposures. Lu et al. used ABM to estimate exposures to NO_2,_ incorporating work location and commuting patterns for the population of the city of Utrecht, the Netherlands. They found very high correlations between residential and mobility-integrated exposures (*R*^2^ > 0.93) [40].

Only a few studies were able to compare personal air pollution measurement data with residential and mobility-integrated exposures. Recently, Wei et al. compared personal measurements of PM_2.5_ and BC for 41 adults in the Netherlands with modeled home-based and mobility-based exposures [41]. They found that mobility-based exposures better represented personal measurements compared to home-based exposures, and that adjusting for the indoor-outdoor ratio was more important than adjusting for travel modes in improving the exposure assessment. A challenge in all direct personal monitoring studies is to assess individual long-term exposure and separation of indoor and outdoor sources. For acute health effect studies, direct personal exposure monitoring has been applied more often [42].

A recent review of air pollution exposure studies comparing residential and mobility-integrated exposures evaluated a number of epidemiological studies that compared health effects using the two approaches to evaluate exposures [17]. They found that the agreement between residential and mobility-integrated exposures was generally high (*R* > 0.8).

### Strengths and limitations

To our knowledge, this is the largest study in terms of participants (*n* = 686), collecting 2 weeks of tracking data with detailed time activity. This allowed for a robust comparison between a number of approaches to determine exposures (residential, mobility-integrated with and without known workplace, real-time activity patterns) and to perform a thorough evaluation.

We conducted the tracking campaigns in two countries, broadening the applicability of our findings to similar demographic and geographical settings.

The participants of the tracking campaigns in both countries, however, were not representative of the full Swiss or Dutch populations, with overrepresentation in the 40–60 year old, higher education, high income and full-time worker groups. The overrepresentation of highly educated participants in the tracking study populations is in agreement with most exposure and epidemiological studies involving invited participation. Participants in both tracking campaigns also resided in more urbanized areas compared to the national population. The travel survey data, which we used to inform the ABM, were, however, specific to each country and more representative, which, given the high comparison between ABM and tracking, suggests that our tracking populations were not too unrepresentative.

Our findings apply to long-term air pollution exposure; we did not evaluate the impact of mobility on short-term exposure estimates. Our findings further apply to the studied pollutants, specifically NO_2_ and PM_2.5_. As NO_2_ is often considered a surrogate for traffic-related pollution, we suspect that qualitatively our findings apply to other traffic-related pollutants. If the spatial pattern of the pollutants differs substantially, however, results may be different.

## Conclusions

Our results suggest that the assessment of air pollution exposures at the residential address in epidemiological studies agrees well with more dynamic exposure assessments integrating time activity patterns based on tracking subjects and/or agent-based modeling. This agreement may be different in settings with different work and commuting patterns. Additionally, given the urban setting of this study, future studies should investigate whether our findings also apply to a more rural setting.

## Supplementary information

Supplementary Figure

## Data Availability

The ABM modeling framework is free and open-source software under the MIT License and available at https://github.com/computationalgeography/agent_based_exposure_assessment.
