# Supplementary material for: Comparison of residential and mobility-integrated air pollution exposures from tracking campaigns and agent-based modelling in Switzerland and the Netherlands
Source: J Expo Sci Environ Epidemiol. 2025 Dec 26;36(3):469–78. doi: 10.1038/s41370-025-00836-5 (PMC13143812; doi:10.1038/s41370-025-00836-5)
Supplement: Supplementary file 1 — Supplementary Figure [file 41370_2025_836_MOESM1_ESM.docx]

Supplementary Figures

Figure S1. Relationship between the ABM and residential exposure, showing the mean of the 50 ABM realizations (row 1) and by drawing a single realization from the 50 ABM realizations randomly from the distribution (row 2). Switzerland (columns 1 and 3) and the Netherlands (columns 2 and 4); NO_2_ (2 left columns), PM_2.5_ (2 right columns). The x=y line is shown as a dashed line.


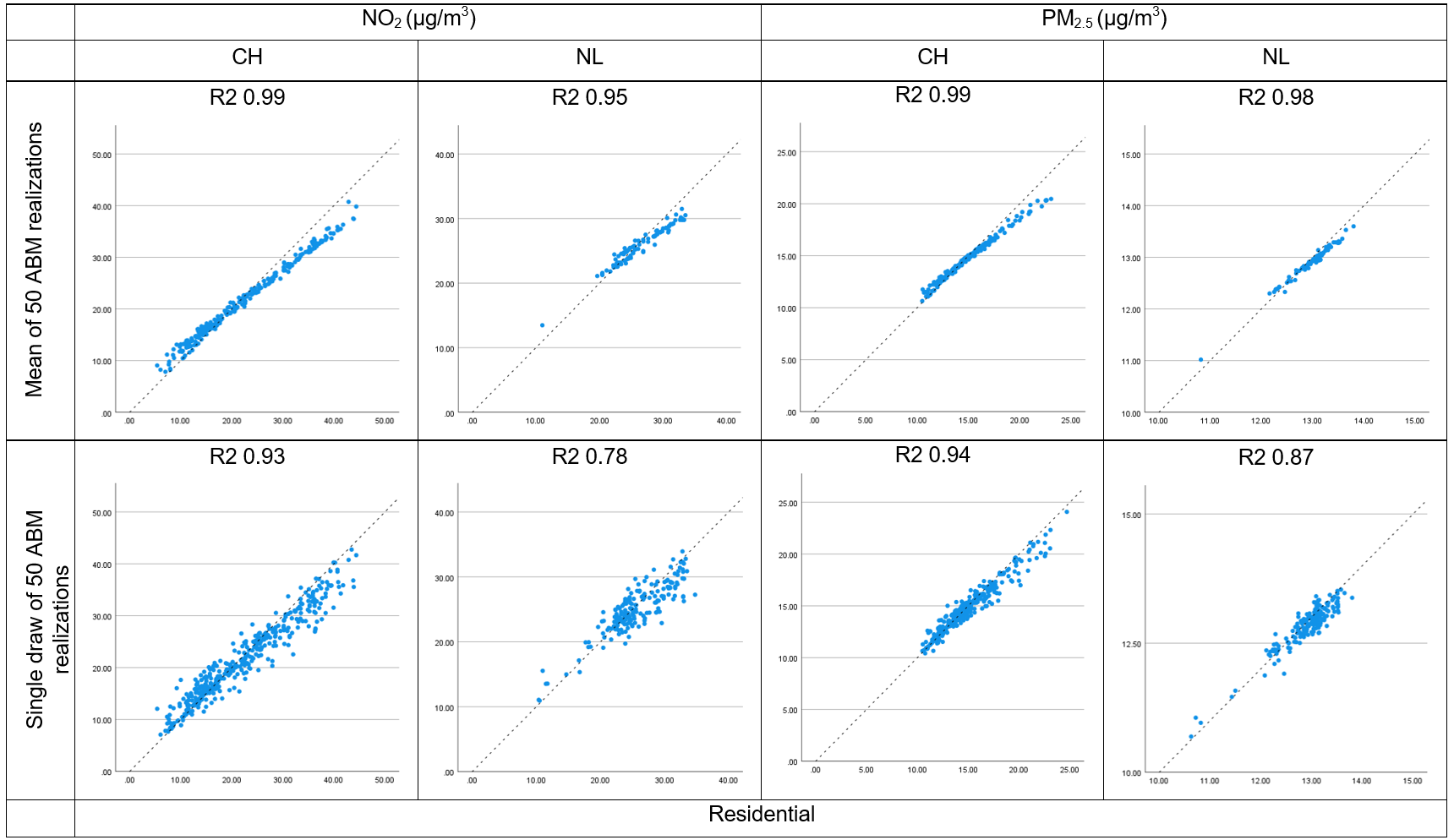


Figure S1. Relationship between the ABM and residential exposure, showing the mean of the 50 ABM realizations (row 1) and by drawing a single realization from the 50 ABM realizations randomly from the distribution (row 2). Switzerland (columns 1 and 3) and the Netherlands (columns 2 and 4); NO_2_ (2 left columns), PM_2.5_ (2 right columns). The x=y line is shown as a dashed line.
